# A Long Distance Phase-Sensitive Optical Time Domain Reflectometer with Simple Structure and High Locating Accuracy

**DOI:** 10.3390/s150921957

**Published:** 2015-09-02

**Authors:** Yi Shi, Hao Feng, Zhoumo Zeng

**Affiliations:** State Key Laboratory for Precision Measurement Technology and Instrument, Tianjin University, Tianjin 30072, China; E-Mails: shy_xflx@163.com (Y.S.); zhmzeng@tju.edu.cn (Z.Z.)

**Keywords:** vibration analysis, scattering measurements, optical time domain reflectometry

## Abstract

A phase-sensitive optical time domain reflectometer (Φ-OTDR) can be used for pipeline security. However, the sensing distance (less than 20 km) of traditional Φ-OTDR is too short for the needs of typical oil and gas pipeline monitoring applications (30–50 km). A simple structure Φ-OTDR system utilizing long pulse, balanced amplified detector and heterodyne detection is proposed in this paper and the sensing range is thereby increased to 60 km. Through analyzing the sensing principle of Φ-OTDR, a novel locating strategy is proposed to maintain the locating accuracy at a few meters when a long pulse (5 µs) is used. The increased pulse width deteriorates the time series of each sensing point seriously. In order to eliminate the deterioration, a data processing technique combining wavelet and empirical mode decomposition is applied in this system. The experiment results show that the sensing distance can be increased to 60 km and the locating accuracy is maintained at 6.8 m.

## 1. Introduction

Distributed optical fiber sensors are those sensors which simultaneously utilize fiber as transmission medium and sensing medium [1]. Because the phase of the transmission light is sensitive to perturbations, the phase-sensitive optical time domain reflectometer (Φ-OTDR) is used for vibration detection application, such as pipeline intrusion and perimeter security.

Oil pipeline monitoring is a tough task because of its long distance and real-time requirements. The early Φ-OTDR-based distributed optical fiber sensing systems can meet the real-time requirement, but their application is limited in the sensing distance, which is only a few kilometers [2]. Valuable works have been carried out to improve the sensing distance. In 2005, Juarez utilized a continuous-wave Er^3+^ doped fiber Fabry-Perot laser and an electro-optic modulator (EOM) to improve the sensing range to 12 km [3]. However, this is still not enough for applications like oil pipeline intrusion detection. Limited by the nonlinear effects in the fiber, it is impossible to increase the peak power of the probe pulses that are launched into the fiber. Then the distributed optical fiber amplifier based on Raman pump or Brillioun pump was applied in Φ-OTDR [4,5,6] to amplify the probe light along the fiber and the sensing distance could be increased to 60 km, and even up to 175 km, but the distributed optical fiber amplifier is complex, expensive, and not suitable for oil pipeline monitoring.

Usually the oil pipeline monitoring demands 30–50 km distance, a few meters locating accuracy and low cost. In this paper, we present a long-distance Φ-OTDR sensing system with 60 km sensing distance, 6.8 m locating accuracy, simple structure and low cost, which is suitable for pipeline security monitoring. In this system, we use long probe pulses, an Erbium-doped Optical Fiber Amplifier (EDFA), balanced detector and heterodyne detection to improve the sensing distance. In order to maintain the locating accuracy, we analyze the process of Φ-OTDR trace generation and propose a novel location strategy. The Φ-OTDR with long probe pulse is also fully discussed in this paper and a suitable signal processing technique which combines wavelet and empirical mode decomposition (EMD) is applied to help figure out the perturbation location.

## 2. Experiment Setup and Principle Analysis

### 2.1. Experiment Setup

**Figure 1:**
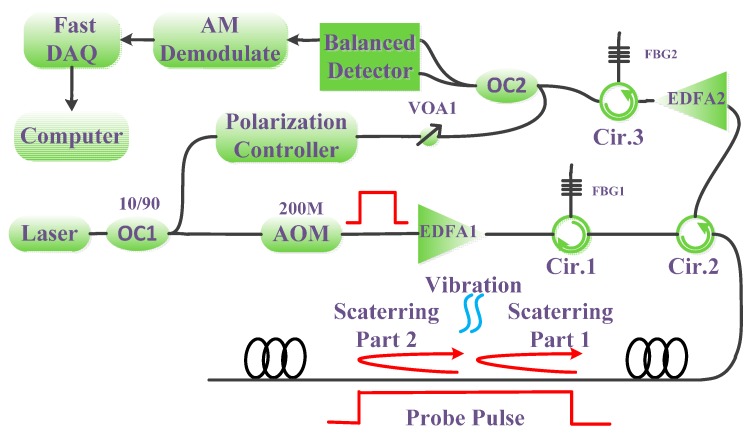
Experimental set up and principle.

The structure of the Φ-OTDR system and its principle are shown in Figure 1. A fiber laser with ultra-narrow line-width (<3 kHz) is used as the optical source. The laser light is divided into two parts, 10% of the light is used as the local light and the other 90% is modulated into pulses with a repetition cycle *T* and pulse width *T_p_* by an acoustic optical modulator (AOM). Then the probe pulses are amplified by an EDFA (amplifier spontaneous emission is filtered by FBG1) and the peak power of probe pulses reaches 200 mW. The probe pulse propagates through the fiber and the Rayleigh backscattering light brings the vibration information along the fiber. The backscattering light from the sensing fiber then meets the local light at optical coupler 2 (OC2) and produces a beat light, whose amplitude contains the information of the backscattering light. Through controlling the local light power, high signal-to-noise ratio (SNR) could be achieved [7]. Then the balanced detector receives the beat light through a differential way, which can suppress the background light and improves the SNR by 3 dB [7]. Through an amplitude demodulation circuit, the useful information can be demodulated and then acquired by a data acquisition card (DAQ).

### 2.2. Parameter Determination

The probe pulse we used in the system is a long pulse compared with the traditional pulse width, which is usually a few hundred nanoseconds. In the OTDR system, the detected Rayleigh backscattering power can be described as [8]:

(1) $$P_{bs}(z)=\left(\frac{1}{2}\right)\gamma P_{0}r\alpha_{s}\nu T_{p}\exp(-2\alpha z)$$

where $P_{0}$ is the peak power of probe pulse at head end, γ is the fraction of capture coefficient of detector, $\alpha_{s}$ is the Rayleigh scattering coefficient, ν is the light speed in fiber, $T_{p}$ is the pulse width and α is the attenuation coefficient.

In order to observe the light at the detector, the light power should be stronger than the detector’s noise. When the signal to noise ratio (SNR) reaches to 1, the input light power is defined as the noise equivalent power (NEP):

(2) $$NEP=P{|}_{SNR=1}$$

Only when the input light power is higher than NEP, the output signal can be recognized. So the maximum sensing distance is:

(3) $$z_{\max}=\left(\frac{1}{2\alpha }\right)\ln(\frac{0.5\gamma P_{0}r\alpha_{s}\nu T_{P}}{NEP})$$

Equation (3) shows that the sensing distance is proportional to the nature log of the pulse width. When the pulse width is improved by *K* = 13.9 dB, the sensing distance will be increased by 46 km (α=0.15dB/km). However, the maximum pulse width is limited by the fiber nonlinear effects, especially the stimulated Brillouin scattering (SBS). The SBS threshold power of continue light is:

(4) $$P_{sbs\_c}=\frac{42}{g_{B}}\frac{A_{eff}}{L_{eff}}\frac{\Delta \nu_{B}+\Delta \nu_{P}}{\Delta \nu_{B}}$$

(5) $$L_{eff}=\left[L-\exp(-\alpha L)\right]/\alpha$$

where $L_{eff}$ is the effective interaction length, $A_{eff}$ is the effective interaction area of fiber, $g_{B}$ is the peak Brillouin gain, $\Delta \nu_{B}\text{​}$is the line width of Brillouin scattering and $\Delta \nu_{P}\text{​}$is the line-width of probe light. When the probe light line-width is much narrower than the Brillouin line-width ($\Delta \nu_{P}\text{​}<<\Delta \nu_{B}$), the SBS threshold power is inversely proportional to the nature log of the total fiber length. When L is 30 km, the $P_{sbs\_c}$ is 2.6 mW. When L is increased to 60 km, the $P_{sbs\_c}$ is declined to 2.06 mW. Because the SBS threshold power is an averaging power, the SBS threshold power of pulse light is:

(6) $$P_{sbs\_p}=P_{sbs\_c}T/T_{p}$$

The maximum input peak power ($P_{0}$) shouldn’t be higher than the SBS threshold power of pulse light ($P_{sbs\_p}$). So we can get the duty cycle of probe pulse is limited by $T_{P}/T<P_{sbs\_c}/P_{0}$ and T>2L/ν. Assuming $P_{sbs\_c}$ is 2 mW and $P_{0}$ is 200 mW, the maximum duty cycle of probe pulse is 1/100.

In order to detect the vibration with more details, the repetition time is designed close to its minimum value (2L/ν). We set the repetition time at 1 ms when the sensing distance is 60 km. Because the maximum pulse width is one percent of the repetition time, the pulse width we used should be less than 10 µs. We choose a 5 µs pulse width (13.9 dB increase compared with 200 ns). With the analysis above, the sensing distance can be increased by 46 km if we choose suitable parameters. The complete determined parameters are shown in Table 1.

**Table 1:** Parameter Determination.

*L* (km)	*P_0_* (mW)	*T* (ms)	*T_p_* (µs)	*∆Z* (km)
60	200	1	5	46

### 2.3. Locating Principle and Strategy

The Rayleigh backscattering trace is formed from multiple scatters. The wave function of the backscattered signal at the input end of fiber can be expressed as [9]:

(7) $$y(t)=\sum_{k=1}^{N}E_{s}(t-\tau_{k})\alpha (\tau_{k})\exp(-\alpha \tau_{k}v)rect(\frac{t-\tau_{k}}{T_{p}})$$

where t is the time, $E_{s}(t)$ is the wave function of the input light wave, N is the total number of scatters in the sensing fiber, $T_{p}$ is the pulse width, α is the fiber attenuation constant, v is the velocity of light in fiber and $\tau_{k}$ as well as $\alpha (\tau_{k})$ are the delay and amplitude of the *k*th scatterer, respectively. The relationship between the delay $\tau_{k}$ and the distance $z_{k}$ from the input end to the *k*th scatterer is $\tau_{k}=2z_{k}/v$. The function $rect(t/T_{p})$ is:

(8) $$rect(\frac{t}{T_{p}})=\{\begin{array}{ll} 1, & while\;0\leq \frac{t}{T_{p}}\leq 1\\ 0, & otherwise \end{array}$$

Thus the optical power is given by:

(9) $$\begin{array}{cc} p(t) & =\langle {\left|y(t)\right|}^{2}\rangle \\ & =\langle \sum_{i=1}^{N}\{I\alpha^{2}(\tau_{i})\exp(-2\alpha \tau_{i}v)rect(\frac{t-\tau_{i}}{T_{p}})\}\\ & \;+2\sum_{i=1}^{N}\sum_{j=i+1}^{N}\{I\gamma_{c}(\tau_{i}-\tau_{j})\alpha (\tau_{i})\alpha (\tau_{j})\\ & \;•\exp(-2\alpha (\tau_{i}+\tau_{j})v)rect(\frac{t-\tau_{i}}{T_{p}})rect(\frac{t-\tau_{j}}{T_{p}})\cos[\omega_{s}(\tau_{i}-\tau_{j})]\}\rangle \\ & =p_{1}(t)+p_{2}(t) \end{array}$$

where 〈〉 represents the average operation, I is the optical power of the input light wave, and $\gamma_{c}(\tau_{i}-\tau_{j})$ is the coherent function of laser. The term $p_{1}(t)$ denotes the sum of the optical power of the backscattered light wave, which is not our interest. The term $p_{2}(t)$ denotes the interference among different scatterers, which results in the jagged appearance of Rayleigh traces and is our interest.

Assume the vibration appears at $z_{m}$, the corresponding scatterer delay is $\tau_{m}=2z_{m}/v$, the optical phase change is $\phi_{m}$ and other parameters won’t be changed by the vibration. Then the difference between Rayleigh backscatter trace at $t_{1}$ without the vibration and at $t_{2}$ with the vibration can be expressed as:

(10) $$\begin{array}{cc} \Delta p_{2}(t) & =p_{2@t_{1}}(t)-p_{2@t_{2}}(t)\\ & =2\sum_{\begin{array}{l} i=1\\ i\neq m \end{array}}^{N}\{I\gamma_{c}(\tau_{i}-\tau_{m})\alpha (\tau_{i})\alpha (\tau_{m})\exp(-2\alpha (\tau_{i}+\tau_{m})v)\\ & \;•rect(\frac{t-\tau_{i}}{T_{p}})rect(\frac{t-\tau_{m}}{T_{p}})\{\cos[\omega_{s}(\tau_{i}-\tau_{m})+\phi_{m}]-\cos[\omega_{s}(\tau_{i}-\tau_{m})]\}\} \end{array}$$

**Figure 2:**
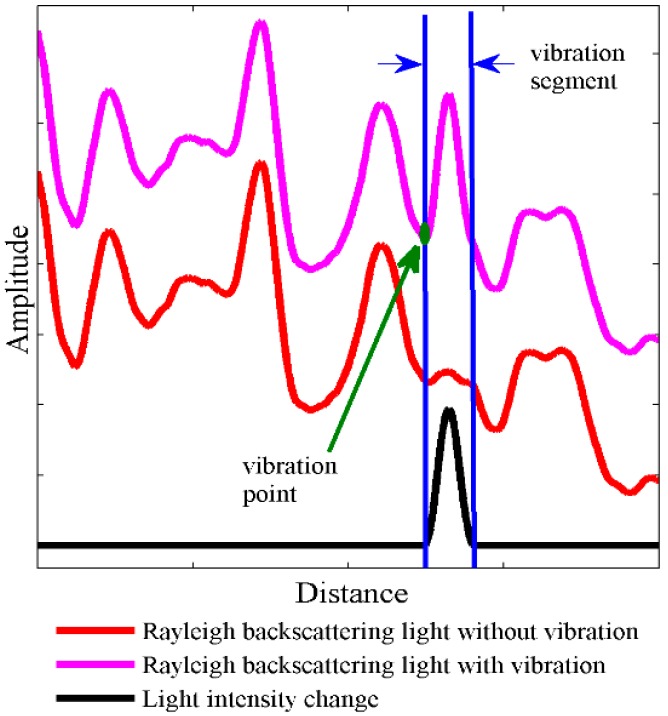
The impact of vibration to the Rayleigh backscattering trace and the relationship between the vibration point and the vibration segment.

According to Equation (10), the non-zero time of $\Delta p_{2}(t)$ is $0\leq \left(t-\tau_{m}\right)/T_{p}\leq 1$. Simplifying the equation we can obtain that the non-zero time is $\tau_{m}\leq t\leq \tau_{m}+T_{p}$ and the corresponding distance is $z_{m}\leq z\leq z_{m}+vT_{p}/2$. This result means the vibration at $z_{m}$ will cause a response segment at $\left[z_{m},z_{m}+vT_{p}/2\right]$. According to Equation (10), the amplitude of light intensity change, $\Delta p_{2}(t)$, will first increase and then decrease, and the shape of the light intensity change is symmetrical. The simulation result of the Rayleigh backscattering light intensity change trace is shown in Figure 2. When a vibration appears at the vibration point, it leads to a light intensity change within the vibration segment, whose length is proportional to the pulse width. Besides, the light intensity change within the vibration segment has a shape which has low sides and high center. Under a noisy condition, the boundary of the response segment might be submerged in noise. Instead, we use the center of the segment to figure out the accurate location, which is expressed as:

(11) $$z=z_{cent}-\frac{T_{p}v}{4}$$

where $z_{cent}$ is the center of the response segment. In order to describe the locating ability of the Equation (11), we use the locating repeatability and locating consistency to define the locating accuracy. The locating repeatability is defined by the maximum location error when the vibration appears at the same place. And the locating consistency is defined by the maximum location error when the vibration appears at different places. These tests will be shown in Section 3.

## 3. Experimental Results

### 3.1. Time Series under Long Pulse and Signal Processing

**Figure 3:**
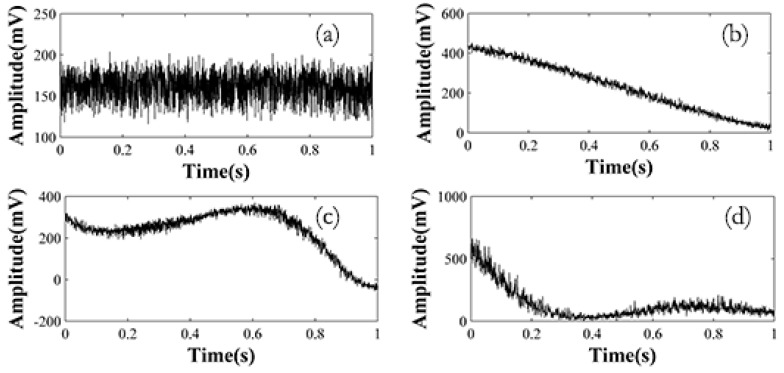
Time series in quiet environment under different pulse widths: (**a**) 200 ns pulse width; (**b**) 1500 ns pulse width; (**c**) 3500 ns pulse width; (**d**) 5000 ns pulse width.

The data picked up from each Rayleigh backscattering trace with the same time delay will form the time series of the corresponding sensing point. With increasing pulse width, the coherent Rayleigh back scattering light becomes more sensitive to phase changes, and the fluctuation are more severe. Figure 3 shows the time series under pulse widths ranging from 200 ns to 5000 ns in a quiet environment.

The time series fluctuate seriously when the pulse width is longer than 200 ns. Because every tiny perturbation within the pulse duration will lead to a fluctuation in the backscattering coherent light, the longer the pulse length is, the easier it is for the time series to be influenced. Thus traditional averaging methods are no longer suitable for the long pulse circumstance. A data processing technique combining wavelet and EMD is proposed to handle it.

The fluctuation is severer when the long pulse is used. It contains low frequencies and is easily influenced by the environmental state. If the fluctuation remains, the locating program will give false locations. In order to avoid false location feedback, we use EMD to decompose the time series into a collection of intrinsic mode functions. The decomposition is expressed as Equation (12). $c_{i}$ is the *i*th intrinsic mode function (IMF) of original signal, which represents the local characteristic signal at different time scales of the original signal, and $\Gamma_{n}$ is the residual error, which represents the global trend of the original signal. Through setting a suitable stopping threshold of EMD process, the fluctuation is preserved in the residual part ($\Gamma_{n}$). Then we can remove the fluctuation and recompose the time series by summating the intrinsic mode functions ($c_{i}$) [10]:

(12) $$X(t)=\sum_{i=1}^{n}c_{i}+\Gamma_{n}$$

The main noise of detector is the shot noise, which can be expressed as:

(13) $$I_{n}={\left[Se(i_{d}+i_{s}+i_{b})M^{2}B\right]}^{1/2}$$

where *S* is a coefficient based on the detector type (for a photovoltaic device *S* = 4), e is the electron charge, $i_{d}$ is the dark current of the detector, $i_{b}$ is the current of the background light, $i_{s}$ is the current of the signal light, *M* is the detector gain and *B* is the frequency band of the detector [11]. The shot noise comes from the background light, signal light and the electronic noise. With the increase of the pulse width, the amplitude of shot noise becomes larger, and it changes with the time series’ fluctuation. When the amplitude of the backscattering light is at a coherent strength state, the amplitude of shot noise is also at a high level. This makes the shot noise difficult to filter by traditional averaging methods or by traditional filtering methods. In order to decrease the time-dependent shot noise, the wavelet denoising method is applied. The wavelet decomposition is expressed as Equation (14):

(14) X(t)=∑i=1nDi+An

$D_{i}$ is the wavelet reconstruction coefficient of high frequency component obtained by the wavelet decomposition of the *i*th layer and An is the reconstruction wavelet coefficient of low frequency component of the *n*th layer. Through wavelet decomposition, the time series are decomposed into different frequency bands. Then the filter threshold will be set according to the shot noise level in each band, respectively [12]. Through removing the shot noise component in each frequency band respectively, the shot noise can be decreased significantly.

**Figure 4:**
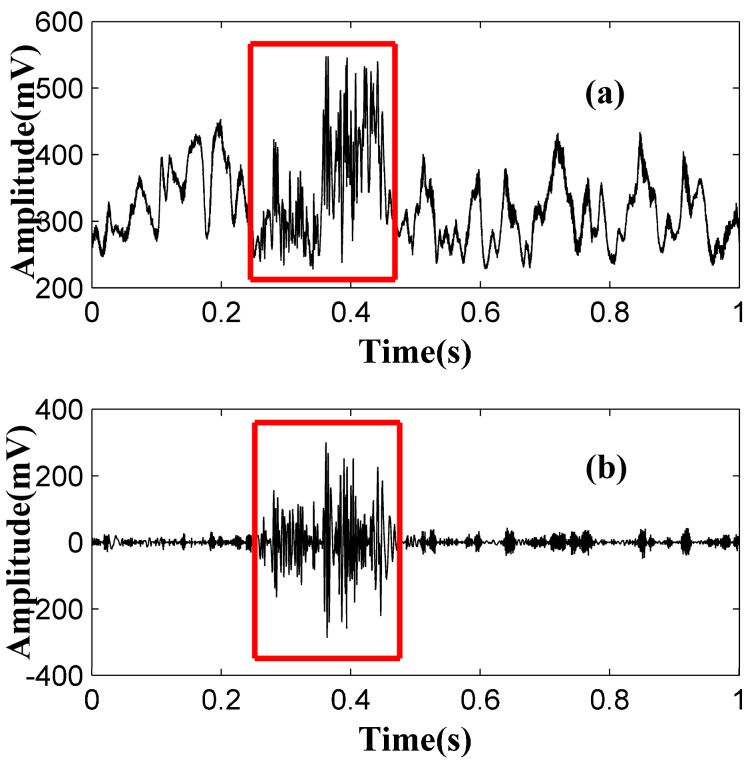
Time series before (**a**) and after (**b**) processing. The red rectangle shows the vibration duration (under 5000 ns pulse width).

The whole data process is described as follows: firstly, the time series are sent to a wavelet denoising program to reduce the short noise. Secondly, the denoised time series are sent to an EMD program to remove the fluctuation. Then we use the peak-to-average ratio (PAR) of the time series to test whether there is a perturbation or not. As the PAR is dimensionless, we can ignore the optical attenuation. If there is a perturbation, the program will figure out the center of the vibration segment and then calculate the location according to Equation (11). Figure 4 shows the performance of the data processing technique under 5000 ns pulse width conditions. Figure 4a shows the time series before processing and the red rectangle shows the duration of the intrusion. With the serious fluctuation and shot noise, it is hard to find out the vibration segment. Without suitable data processing, it will also cause false alarms. Figure 4b shows the data after processing. We can see that the shot noise and data fluctuation are reduced greatly both in the background part (0.5 s–0.9 s) and in the vibration part (0.2 s–0.5 s). Besides, the details of vibration are kept, which is used for the following data analysis, like event recognition.

### 3.2. Spatial Trace and Vibration Segment

**Figure 5:**
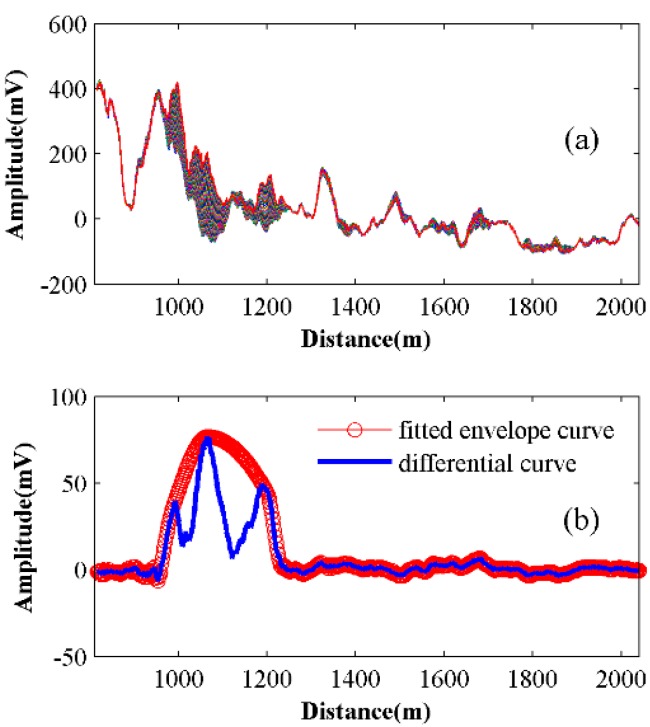
(**a**) Fifty after-averaged traces superimposed with amplitude change from 970 m to 1270 m. (**b**) Differential trace (the blue solid line) and its fitted envelope curve (the red circular line).

In order to show the characteristic of the spatial traces under long pulse conditions, 50 after-averaged traces, which come from 100 original Rayleigh backscattering traces with a moving average number 50, are illustrated in Figure 5a. The pulse width is 3000 ns and the repeat rate of the pulse is 5 kHz. An artificial knock acts as the vibration source at about 970 m. As the 100 original traces only take up 0.02 s, the background fluctuation is weak and only wavelet denoising is applied. We can observe a clear amplitude change from about 970 m to 1270 m, corresponding to the artificial knock at 970 m under a 3000 ns pulse width. The blue solid line in Figure 5b is the difference between each trace with an interval number 10. There are two valleys between 970 m and 1270 m. This is due to the destructive interference problem [13], which leads to certain places being insensitive to vibration.

When the phase part $\omega_{s}(\tau_{i}-\tau_{m})$ in Equation (10) is kπ(k=1,2,3...), the sensor will be insensitive and the light intensity change will be small. When the phase part is kπ+π/2(k=1,2,3...), the sensor will be sensitive and the light intensity change will be large. In order to figure out the vibration segment, the envelope fitting is applied, which is the red circular line in Figure 5b. The fitted envelope curve shows low-sides and a high-middle shape, which matches Figure 2. Through a threshold method, the vibration segment can be figured out.

### 3.3. Repeatability Tests under Different Pulse Widths

**Figure 6:**
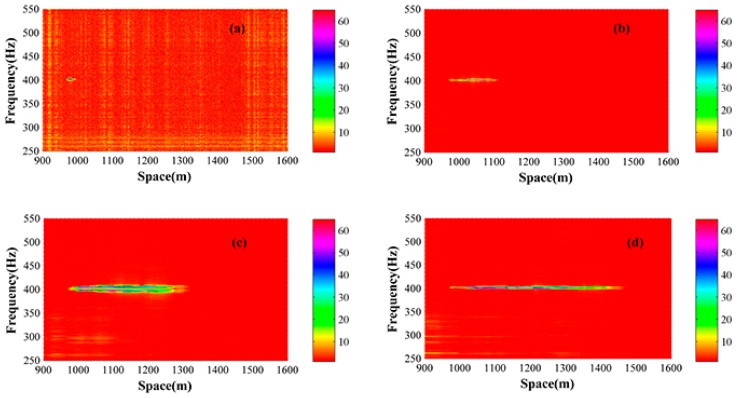
Frequency and space relationship under different pulse widths. (**a**) 200 ns pulse width; (**b**) 1500 ns pulse width; (**c**) 3500 ns pulse width; (**d**) 5000 ns pulse width.

**Figure 7:**
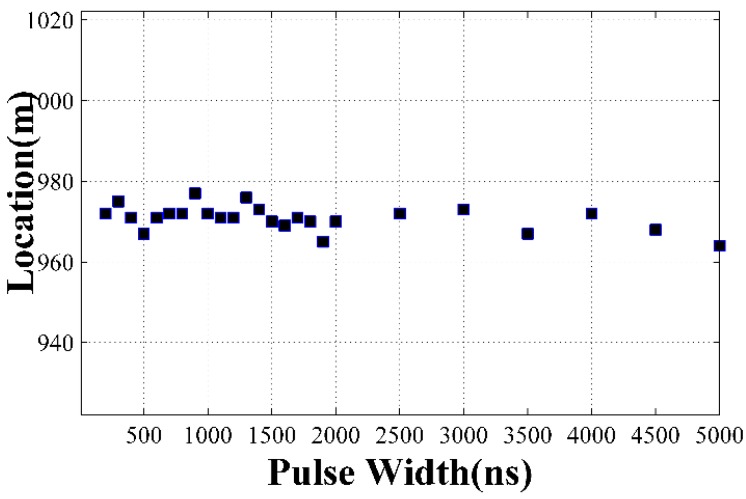
Locating results under different pulse widths.

A piezoelectric transducer (PZT) tube is set at 970 m to act as the vibration source and a 400 Hz sinusoidal wave from a signal generator is used to drive the PZT tube. The sample frequency is 50 MHz, corresponding to 2 m spatial resolution. Different pulse widths ranging from 200 ns to 5 µs are applied and then a program calculates the location automatically. In order to figure out the vibration segment accurately, the time series of every sample point is transformed to frequency domain, and obvious value can be found at 400 Hz place. Figure 6 shows the data under 200 ns, 1500 ns, 3500 ns and 5000 ns pulse width conditions and the start and end points of the vibration segment can be figured out through a threshold method. Table 2 shows the results under different pulse widths and Figure 7 shows their relationship. The average location is 970.8 m, which is used as the true location, and the maximum error is 6.8 m, which appears under the 5000 ns pulse width.

**Table 2:** Locating results under different pulse widths.

Pulse Width (ns)	Start Point (m)	End Point (m)	Location (m)
200	970	994	972
300	970	1010	975
400	972	1010	971
500	968	1016	967
600	970	1032	971
700	970	1044	972
800	970	1054	972
900	972	1072	977
1000	970	1074	972
1100	972	1080	971
1200	968	1094	971
1300	976	1106	976
1400	974	1112	973
1500	970	1120	970
1600	970	1128	969
1700	970	1142	971
1800	970	1150	970
1900	968	1152	965
2000	972	1168	970
2500	972	1222	972
3000	972	1274	973
3500	970	1314	967
4000	972	1372	972
4500	970	1416	968
5000	970	1458	964

### 3.4. Repeatability Tests under Constant Pulse Width

**Figure 8:**
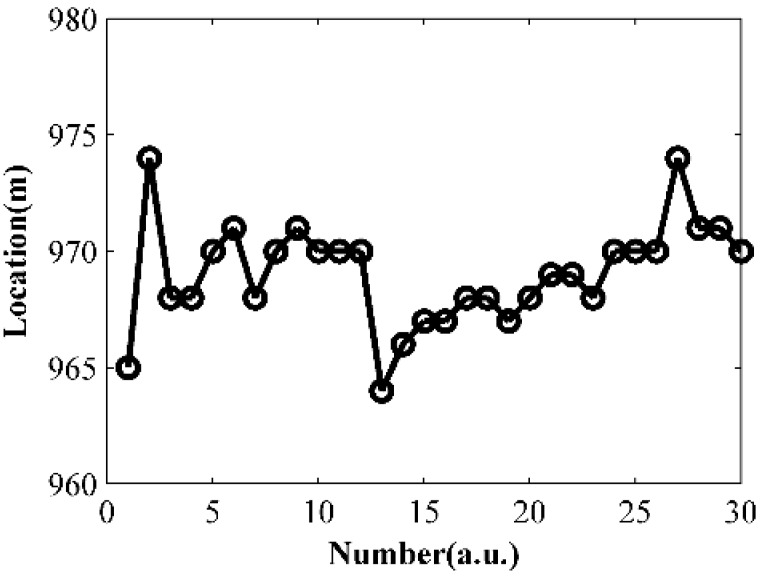
Locating results under 5000 ns pulse width.

In order to test the repeatability of the system, we repeat the test in Section 3.3 at the same point 30 times under 5000 ns pulse width and 50 MHz sample frequency. The results are shown in Figure 8. We use the average location from Section 3.3 as the true location. The maximum error then is 6.8 m and the standard error is 2.2 m.

### 3.5. Different Perturbation Points under Long Pulse Conditions

**Figure 9:**
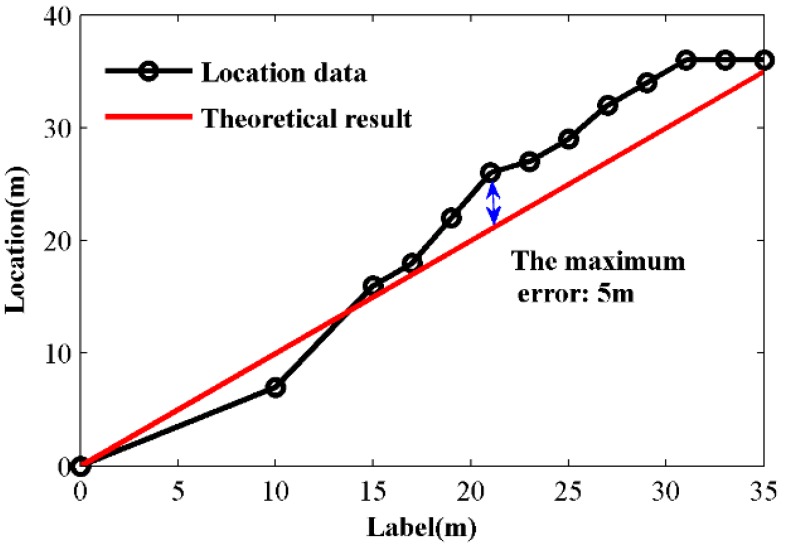
Average locating result under different perturbation points (the black line is the locating result and the red line is the reference line).

In order to test the locating consistency of the system, a test of perturbations at different places labeled from 0 m to 35 m is carried out. Here, because it is hard to obtain the accurate absolute distance on the fiber, we use the relative distance as the true value. The test results are also relative distances obtained by subtracting the location of the 0 m test point. With a 50 MHz sample frequency, the distance between each sample point is 2 m. Hence, the system can response a different perturbation with a 2 m interval theoretically. Figure 9 shows the average locating results of each perturbation point and the maximum error is 5 m, where the applied pulse width is 5 µs. Combined with the tests in Section 3.3 and Section 3.4, the locating accuracy of the system is 6.8 m.

**Figure 10:**
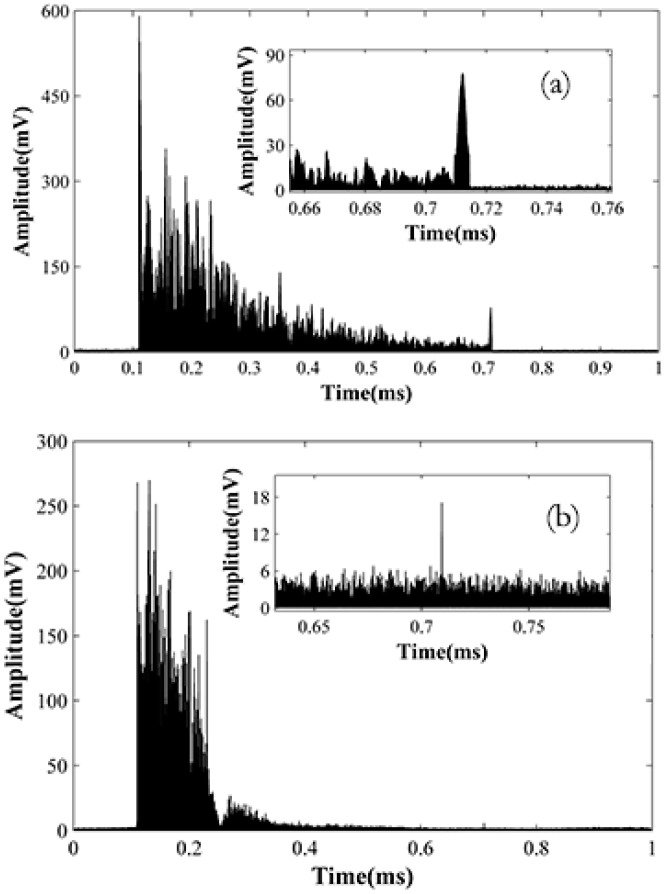
Back scattering traces and their details under 5000 ns (**a**) and 200 ns (**b**) pulse width.

### 3.6. Tests of 60 km Sensing Distance

The test system is shown in Figure 1. We connect three fiber coils of 12, 24 and 24 km together to get a 60 km test fiber. The pulse width is 5000 ns and repetition time is 1 ms. The EDFA before the detector is used to compensate the loss of fiber connectors. The balanced detector is used to enhance the SNR. Figure 10a shows the backscattering trace after demodulation. The start of the trace is about 0.11 ms and the end of the trace is about 0.71 ms. The length of the trace is 6 ms, corresponding to 60 km in fiber. Figure 10b is the trace of 200 ns pulse for comparison. The insert figures in Figure 10 are the details at 60 km place. From Figure 10, we can see the 5000 ns trace still has about 25 mV intensity at 60 km and the background noise is only about 5 mV. Usually, the signal with SNR > 2 dB can be useful. Thus the signal quality is enough for the location program. In the other aspect, the 200 ns trace only shows an end reflection at 60 km and the Rayleigh backscattering light is submerged in the noise.

## 4. Discussion

The system is developed for oil and gas pipeline monitoring. Usually, the average distance between two transfer stations is 30–50 km and the main perturbation is human intrusion, such as digging. The tests above show that our system can meet these requests.

Compared with the ones utilizing a distributed Brillouin amplifier or a distributed Raman amplifier, our system is easier to achieve, less costly and more reliable. Besides, the system doesn’t need an outdoor relaying section, which is also an advantage for the long distance pipeline monitoring.

The former pipeline monitoring system used in the field is the one based on two Mach-Zehnder interferometers [14]. It has a sensing distance of 50 km and a locating accuracy of a hundred meters. Compared with that, our system has obvious superiority in locating accuracy and a similar sensing distance. What’s more, our system also has capacity to monitor multiple disturbances with long intervals at the same time.

In some cases, the vibration source may be strong, such as a bomb explosion, and a section of fiber will be influenced at the same time. If so, the vibration section in the result curve will be longer than a conventional one. For example, if the probe pulse length is 200 m and the fibers from 30.0 km to 30.5 km are influenced by a bomb explosion, the vibration section in the result curve will be from 30.0 km to 30.7 km. Thus, if the vibration section is much longer than the pulse length, we may find that there is a strong vibration and the start of the vibration section will show the place of the strong vibration event.

## 5. Conclusions

In this paper, we demonstrate a long distance Φ-OTDR with high locating accuracy and simple structure, which is quite suitable for pipeline monitoring. We analyze the model under long pulse conditions, propose a novel locating strategy and find suitable data processing techniques. Finally, long pulse, heterodyne detection, a receiving end EDFA, balanced amplified detector and data processing technique combining wavelet and EMD are utilized to ensure the signal SNR and locating accuracy over a 60 km fiber. The tests show that the locating accuracy of the system is 6.8 m.
